# T Cell Memory in the Context of Persistent Herpes Viral Infections

**DOI:** 10.3390/v4071116

**Published:** 2012-07-20

**Authors:** Nicole Torti, Annette Oxenius

**Affiliations:** Institute of Microbiology, ETH Zurich, CH-8093 Zurich, Switzerland; Email: torti@micro.biol.ethz.ch

**Keywords:** herpes virus infection, CD8 T cells, memory inflation: secondary lymphoid organs, peripheral tissues, CD8 T cell function, CD8 T cell maintenance, CD8 T cell phenotype

## Abstract

The generation of a functional memory T cell pool upon primary encounter with an infectious pathogen is, in combination with humoral immunity, an essential process to confer protective immunity against reencounters with the same pathogen. A prerequisite for the generation and maintenance of long-lived memory T cells is the clearance of antigen after infection, which is fulfilled upon resolution of acute viral infections. Memory T cells play also a fundamental role during persistent viral infections by contributing to relative control and immuosurveillance of active replication or viral reactivation, respectively. However, the dynamics, the phenotype, the mechanisms of maintenance and the functionality of memory T cells which develop upon acute/resolved infection as opposed to chronic/latent infection differ substantially. In this review we summarize current knowledge about memory CD8 T cell responses elicited during α-, β-, and γ-herpes viral infections with major emphasis on the induction, maintenance and function of virus-specific memory CD8 T cells during viral latency and we discuss how the peculiar features of these memory CD8 T cell responses are related to the biology of these persistently infecting viruses.

## 1. Introduction

In the context of immunity, the notion of ‘memory’ refers to a property of the adaptive immune system to specifically remember and recognize pathogens that have already infected the host in the past and to mount faster and stronger responses each time the same pathogen is encountered. Since the discovery of vaccination and passive immunization in the late 19^th ^– early 20^th^, the ability to memorize pathogens and confer protection has been for long time attributed uniquely to antibody-producing cells; it was only half century later, in the mid-60s, that Jacques Miller suggested that there was not just one but two subsets of lymphocytes, a bone-marrow-dependent antibody-producing cells and a thymus-dependent helper cell that were called T cells [1,2]. Since then, many facets of T-cell functions at steady-state and during an immune response have been characterized, and, by the end of the 80s, the notion of T cell memory began to develop [3,4]. Today we have a broad understanding on how T cell memory is generated during the course of an infection, how this is preserved throughout the life of the host and how this confers protection upon secondary infections. In this review, we will focus on memory CD8 T cells and how they adapt to the type of pathogen and to the anatomical localization to provide the host with best protection for any type of infection. In particular, we will focus our attention on the development and functional programming of memory CD8 T cells during persistent/latent viral infections, raising questions as to how continuous antigen stimulation shapes the memory CD8 T cell compartment, how the anatomical microenvironment of antigen presentation influence the state of memory and what role these memory CD8 T cells have in controlling viral latency.

### 1.1. T Cell Memory: More than Just ‘Central’ or ‘Effector’

The fact that memory CD8 T cells formed upon the resolution of an infection have a degree of heterogeneity in terms of anatomical localization, function and phenotype began to emerge already in the nineties, as two major subtypes were identified: central-memory T cells (T_CM_), and effector-memory T cells [5,6,7,8,9]. This distinction was made originally according to the different anatomical localization of memory T cells, with T_CM_ localizing to secondary lymphoid organs due to high expression of the lymph node homing markers CD62L and CCR7, and T_EM_ being excluded from lymph nodes because they fail to up-regulate these cell-surface receptors, and are therefore restricted to peripheral tissues [8,10,11]. Further analysis of cytokine receptor expression patterns underlined substantially different survival requirements between T_CM_ and T_EM_. In fact, memory T cells in secondary lymphoid organs have a considerably higher expression of IL-7 and IL-15 receptors compared to memory T cells in the periphery [12,13]. The identification of IL-7 as ‘survival chemokine’ and IL-15 as key regulator of homeostatic turnover of memory T cells, implied that T_CM_ were long-lived and stably maintained upon clearance of the pathogen, whereas T_EM_ persistence in the periphery was generally believed to rely on cognate antigen stimulation because of their poor responsiveness to these γc cytokines [14,15,16,17,18,19,20]. However, this notion has lately been challenged and a far higher complexity of maintenance for memory T cells in non-lymphoid tissues is starting to emerge [21]. Recent work has revealed for example that in response to localized infections such as by Herpes Simplex Virus (HSV), Lymphocytic Choriomeningitis Virus (LCMV), Vaccinia Virus (VV) or Vesicular Stomatitis Virus (VSV), a distinct population of non-recirculating memory T cells expressing CD69 and CD103 infiltrate mucosal sites but also other organs such as the skin and the brain, and persist in the absence of antigen stimulation for several months, or even years [22,23,24,25,26,27]. These so called tissue-resident memory T cells (TRM) have little or absent homeostatic turnover and their persistence has been shown to be intimately dependent on CD103 interacting with E-cadherin expressed on epithelial cells. Also the interaction with extracellular matrix, but in this case mediated by VLA-1 binding to type IV Collagen in the lung airways, has been shown to promote prolonged survival and persistence of antigen-specific T_EM_ after clearance of Influenza infection [28]. Moreover, prolonged persistence of memory CD8 T cells upon influenza clearance is also sustained by IL-15 being cross-presented by pulmonary pDCs [29]. These tissue-specific behaviors of T_EM_ cells indicate that the tissue environment has a strong influence on the phenotype, function and maintenance of the developing memory T cell population [30]. Thus, referring to ‘peripheral T_EM_’ is not adequate; memory T cells in the periphery are characterized by a high degree of heterogeneity in phenotype, function, survival requirements, all factors being intimately linked to the nature of the insulting pathogen, the route of the infection, and the anatomy and function of the resident tissue. In addition, other factors such as the strength, duration and timing of interaction between TCR and peptide-MHC, the induction of pro- or anti-inflammatory environments, the precursor frequency of antigen-specific CD8 T cells, the cellular tropism of a pathogen and its persistence in the host, are all influencing the composition and maintenance of memory T cell compartment [31,32,33,34,35,36]. In particular, repetitive antigen stimulation has been shown to alter dramatically the behavior of memory T cells. In fact, secondary memory T cells have been shown to be more protective than primary memory T cells in response to an acute viral infection, to have prolonged survival in the periphery and to be slower in up-regulating CD62L [37,38]. The alteration of T cell function and homeostasis is much more drastic in situations of persistent infections, where memory T cells repeatedly encounter their cognate antigens, precluding the development of long-lived central memory T cells and sometimes resulting in large expansion of these cells, or in some cases, in a compromised functionality and even in their physical deletion [39,40,41,42,43,44,45,46]. The severity of this perturbed T cell behavior is highly dependent on the amount of antigen exposure. For instance, high dose infection with LCMV induces a chronic productive infection which might last for months or even forever, causing repetitive stimulation of antigen-specific CD8 T cells. These cells are present at high numbers in infected hosts but fail to survive when transferred into a naïve host, indicating that their maintenance and local proliferation is antigen-dependent [47]. A consequence of this repetitive TCR stimulation is an impaired NFAT translocation to the nucleus, which results in a gradual loss of T cell effector functions (compromised IL-2 production first, followed by TNFα and finally IFNγ secretion), a phenomenon which has been referred to as ‘T cell exhaustion’ [39,48,49,50,51,52,53]. 

A different situation is observed during latent viral infections, where T cells remain functional and provide active immune surveillance at the sites where latency is established. It is difficult to estimate the overall antigen burden during latency since in most cases this falls below the detection limit. It is assumed that a low-level of persistent infection co-exists with true latency and is the result of rare events of viral reactivations and shedding which happen stochastically throughout the life of the infected host. The frequency and the mode of memory CD8 T cell stimulation during latency might not be sufficient to impair their functionality [54,55,56] as seen for chronic LCMV infection, but enough to induce sometimes impressive CD8 T cell expansions.

Prototypes of latent infections are infections with herpes viruses, which include eight viruses that infect humans and are classified into α-, β- and γ subfamilies. Human herpes viruses have co-evolved over millions of years with their hosts and are therefore exquisitely adapted to them. These viruses establish an asymptomatic latent infection, whereby both anatomical and cellular sites of latency establishment are distinct between various members of herpes viruses [57,58]. There is clear evidence that T cell immunity is critically involved in controlling primary and latent infections, and a growing body of evidence indicates that peripheral memory T cells of either the T_EM_ or T_RM_ subtypes play a key role in immune surveillance during latent viral infections, by sensing and immediately terminating viral reactivation events from latently infected cells [22,59,60,61,62,63,64]. Therefore, it might be of interest for the immune system to maintain a pool of T_EM_ cells in close proximity to latently infected cells, which are readily activated and terminate recurrent viral reactivation events by virtue of their rapidity in producing effector cytokines such as IFNγ and TNFα. Indeed, the induction of a strong T_EM_ response is a peculiarity of herpes viruses, whereby a sum of factors such as the route of infection, the site of CD8 T cell priming, the duration of acute infection and especially the site, both anatomical and cellular, of latency establishment, determine the size and the phenotype of the CD8 T cell memory pool [65,66]. 

In the following sections, memory T cell responses induced against selected members of the α-, β-, and γ-herpes virus families will be discussed in a comparative manner, with major focus on the tissue-specific mechanism(s) of maintenance of the peripheral memory pool during latency. 

## 2. Memory Response against β-Herpesviruses: Murine Cytomegalovirus (MCMV)

### 2.1. CD8 T Cell Immunity to CMV

**Figure 1:**
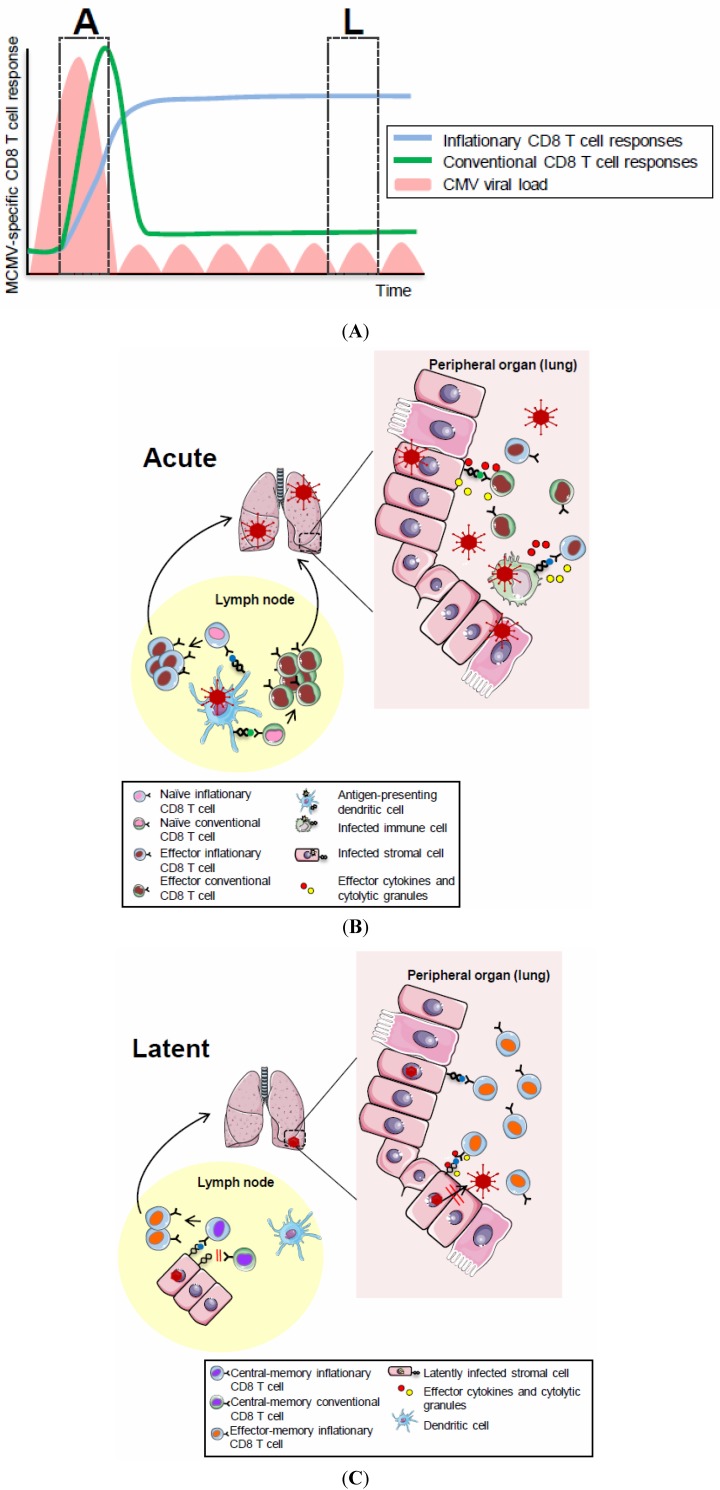
(**A**) CD8 T cell dynamics during acute and latent MCMV infection. The acute phase (“A”) of MCMV infection is characterized by lytic viral replication (pink areas) which is controlled by NK and T cells with an organ-specific kinetics. The following phase of latent infection (“L”) is thought to be associated with local viral reactivation events which are readily controlled by MCMV-specific immunity. CD8 T cell responses specific for the majority of MCMV-derived epitopes follow a classical expansion, contraction and memory phase (green, conventional response) while CD8 T cells with some MCMV-specificities undergo memory inflation (blue, inflationary response), eventually stabilizing at high numbers with an activated phenotype. (**B**) CD8 T cell priming in secondary lymphoid organs. Priming of conventional and inflationary MCMV-specific CD8 T cell responses occurs in secondary lymphoid organs and largely depends on the presence of cross-presentation competent dendritic cells. Activated MCMV-specific CD8 T cells, equipped with effector functions, exit secondary lymphoid organs and home to peripheral tissues where they contribute to control of lytic MCMV replication; (**C**) Memory CD8 T cell inflation during latent MCMV infection. Inflationary MCMV-specific CD8 T cells accumulate in peripheral tissues where they are maintained at high numbers with an effector memory phenotype. This memory CD8 T cell inflation is triggered by MCMV antigen recognition on latently infected non-hematopoietic cells within lymph nodes where inflationary CD8 T cells exhibiting a central memory phenotype are restimulated to proliferate, to acquire effector phenotypes and to migrate into peripheral tissues where they contribute to local immune-surveillance of latent/reactivating MCMV infection.

CMV seropositivity in humans is associated with a massive pool of CMV-specific memory CD8 T cells, especially in the elderly, occupying ~10% of the circulating memory compartment [67]. Phenotypically, these cells are mostly CCR7^−^, CD27^−^ and CD28^−^, all indicators of the T_EM_ subset of memory cells, and the majority show high expression of CD57, which has been associated with replicative senescence [68,69]. In addition, CMV-specific CD8 T cells might also re-express CD45RA, indicative of terminally differentiated effector cells. Functionally, CMV-specific CD8 T cells secrete MIP-1β and TNFα, and to a lower extent perforin upon *ex-vivo* peptide stimulation and they show immediate cytotoxicity [68]. Similarly, naturally infected mice as well as laboratory mouse strains latently infected with MCMV develop a very large population of CD8 T_EM_ cells in the spleen, but also in peripheral organs such as the lungs and the liver [56,70,71,72,73,74,75]. The identification of the whole spectrum of MHC-I restricted epitopes eliciting a CD8 T cell response in C57BL/6 mice made it possible to perform longitudinal analyses of the different responses. This work revealed that two very distinct kinetic patterns of CD8 T cell responses are induced upon infection with MCMV. The majority of the CD8 T cells, referred to as ‘conventional CD8 T cells’, undergo expansion during the acute phase of infection followed by rapid contraction, eventually resulting in low numbers of memory cells that re-express CD62L and migrate to secondary lymphoid organs, where they are stably maintained throughout the latent phase of infection by cytokine-induced homeostatic proliferation. In contrast, at least five epitopes (M38_316–323_, m139_419–426_, IE3_416–423_, IE3_461–475_, M102_486–500_) follow a so called ‘inflationary’ response, characterized by continuous expansion even after control of acute lytic infection, to eventually stabilize at high percentages during latency [75] (Figure 1A). As observed for HCMV-specific CD8 T cells, inflationary CD8 T cells in mice display the classical phenotype of terminally differentiated T_EM _cells in the periphery (CCR7^−^ CD62L^−^ IL7Rα^−^ CD27^−^ CD28^−^ KLRG1^+^), and retain their cytotoxic functions as well as the ability to secrete IFNγ and TNFα [56,76]. 

### 2.2. Mechanism of CD8 T Cell Inflation During CMV Infection

The mechanism by which CMV induces such a strong memory response during latency is beginning to be understood but is far from being resolved. The T_EM _phenotype of the ‘inflationary’ cells strongly suggests that repetitive antigen exposure is the major driver of their accumulation and maintenance at high percentages during latency, implying an ongoing transcriptional activity of the virus during latency. This is indeed what occurs, as CMV transcripts have been detected in the lungs of latently infected mice, albeit at a very low rate and in a stochastic manner [61]. But why would only a minority of the MCMV-specific CD8 T cells that participate in the acute response be repeatedly stimulated, while others do not? One possible explanation for the existence of two different CD8 T cell kinetics is that some viral genes are more abundantly expressed than others during latency. Most of the work in this respect has been done in the Balb/c mouse strain, where highly sensitive RT-PCR detected immediate-early 1 (IE1) and IE2 transcripts, without detecting any early or late gene-products [77]. This might explain the immunodominance of the IE1-derived pp89 epitope in latently infected Balb/c mice. According to a model called the “*immune sensing hypothesis of latency control*”, presentation of the pp89 epitope by cells initiating viral reactivation will stimulate IE-1 specific CD8 T cells, which would in turn terminate the ongoing reactivation event, providing a strong indication that this large pool of T_EM_ cells in the periphery is an integral part of the immune-surveillance to suppress CMV reactivation. Consistent with this, mice infected with a MCMV mutant strain carrying a mutation in the pp89 epitope which abrogates MHC class I binding show a 5-fold increase in IE1 transcripts with transition to IE3 transcripts in latently infected lungs, though transcription does not proceed to late genes [61]. 

Currently, nothing is known about the expression profile of M38, m139, IE3 and M102 genes in latently infected C57BL/6 mice. Our group has tried to detect differences in the expression levels of M38 and M45 genes during latency (the first one inducing an inflationary, the second one a conventional response) by qRT-PCR, but we were unable to detect any viral transcripts during latency, and during the acute phase of infection the two genes were expressed at similar levels (unpublished data). 

While it remains an open question whether a preferential viral gene expression profile determines the specificities of inflating CD8 T cell populations during latency, it is possible that additional mechanisms are involved, as suggested by the observation that a second epitope contained in the M38 gene product, M38_38–45_, induces a conventional CD8 T cell response [74]. Hence, it is possible that post-transcriptional mechanisms are involved, for example at the antigen processing level. In this line, it has recently been reported that conventional CD8 T cells display a higher dependence on the immunoproteasome subunit LMP7 compared to inflationary CD8 T cells [78]. The immunoproteasome is constitutively expressed by immune cells like DCs and macrophages, whereas expression in non-immune cells can be induced by IFNγ. As there is increasing evidence that non-hematopoietic cells, particularly endothelial cells, are a major site of MCMV latency [79,80,81,82], it is an attractive hypothesis that these cells (which are limited to antigen processing by the constitutive proteasome by virtue of their lack of the immunoproteasome during viral latency when IFNγ levels are low) would only be able to process and present those antigens whose processing is independent of the immunoproteasome. 

A series of recent findings and observations are supporting the hypothesis that non-immune cells are indeed the key antigen-presenting cells during CMV latency. Firstly, contrary to priming of the MCMV-specific CD8 T cell response which relies on cross-presentation [83,84], accumulation of CD8 T cells during latency is independent of the major subsets of cross-presenting DCs [84]. The dependence on cross-presentation for priming finds a rational explanation in the numerous immune evasion strategies that CMV uses to interfere with nearly every step of the antigen-presentation machinery of the infected cell [85,86,87,88] (Figure 1B). The fact that priming and inflation have different requirements for cross-presentation might indicate that immune evasion is less effective during latency, or that it inefficiently targets the antigens that will ultimately induce inflation. A very interesting observation was that memory inflation was not impaired when a spread defective virus was used or when virus replication was selectively blocked before or after MCMV infection, implying that productive viral replication is not a prerequisite for memory inflation [89]. Combining these data, a model can be drawn where a so far undefined population/s of non-hematopoietic cells that get infected already at the first round of infection will become a major reservoir of latent CMV, and will occasionally process and present a limited and defined set of CMV-derived antigens to memory CD8 T cells, causing their accumulation or/and maintenance in the periphery (Figure 1C). In support of this hypothesis, using a chimeric system in which the expression of the H-2K^b ^molecule was restricted to immune-cells, we recently showed that inflation of the H-2K^b^-restricted M38-specific CD8 T cell response was completely dependent on antigen presentation by non-hematopoietic cells [82]. Indeed, despite robust priming, memory inflation was abolished in these mice and the otherwise highly activated inflationary CD8 T cells displayed a T_CM_ phenotype during latency. 

This finding gave rise to a series of new questions: is antigen-presentation by non-hematopoietic cells required all over the time or only for the induction of the inflationary response? How do these cells respond to antigen stimulation by ‘commonly week antigen-presenting cells’ as non-hematopoietic cells are? How is the number of T_EM_ cells in the periphery kept at a constant number during latency? Inflationary CD8 T cells divide only sporadically during latency in peripheral tissues such as the lung and the liver, and they do not express CD69 [56,72,73,90], suggesting that events of antigen encounter are either extremely rare in the periphery, absent, or that non-hematopoietic cells lack the appropriate costimulatory molecules to induce division of the CD8 T cells responding to antigen stimulations. Therefore, it came to a surprise that inflationary CD8 T cells decayed with the same kinetics when transferred into infected or naïve hosts, with a half-life of about 40 days, although a low degree of antigen-driven proliferation was detectable [56,82].

This finding has two basic implications: first, if the T_EM_ pool in the periphery is not self-sustainable by means of antigen-driven proliferation, it must be constantly filled with new cells [65]. And second: if is not antigen-mediated, which signals promote T_EM_ survival in the periphery? A hint to the first point came from the observation that whereas inflation of M38-specific CD8 T cells was seen in peripheral blood and in organs such as the lungs and the liver, it was not observed in the lymph nodes, where their numbers constantly decreased upon control of the acute infection and possessed largely a T_CM_ phenotype [82]. Notably, the presence of a small-population of central-memory CMV-specific CD8 T cells in the lymph-nodes was also recently confirmed in humans [91]. We found that in contrast to terminally differentiated T_EM_ in the periphery, T_CM_ cells from the LNs proliferate extensively when transferred into infected hosts but not when transferred into naïve hosts, providing a strong indication that the source of newly activated effector cells filling the peripheral inflationary pool was in the lymph nodes. Notably, local proliferation of inflationary T_CM_ cells during viral latency in the lymph nodes was strictly dependent on antigen presentation by non-hematopoietic cells. Based on these findings, our current model of maintenance of the inflationary pool foresees that viral reactivation events in non-hematopoietic cells in the lymph nodes are sensed by locally resident T_CM_ cells, resulting in their extensive proliferation and differentiation into T_EM_ cells, which can consequently migrate and home to peripheral tissues. In such a scenario, the long-term maintenance of peripheral T_EM_ cells being able to perform constant immune-surveillance duties is guaranteed as it is based on recurrent tapping of T_CM_ resources from the lymph nodes. 

### 2.3. Survival Mechanisms of CMV-specific Memory T Cells in the Periphery

The second issue that emerged from the observation that persistence of T_EM_ cells in the peripheral organs is antigen-independent was about their survival strategy. Before starting a discussion about the factors that might promote prolonged survival of MCMV-specific memory T cells in peripheral organs, it is legitimate to question whether a half-life of 40 days has to be considered ‘normal’ for T_EM _cell, or particularly long and perhaps caused by a CMV-intrinsic pro-survival mechanism. T_EM_ are per definition believed to be unstable without their cognate antigen, but exhaustive studies aiming at determining their longevity are still poor. In a study performed by Kaech et al, when IL7R^hi^ and IL7R^lo^ cells isolated at day 15 after infection with LCMV where transferred into naïve recipients, IL7R^hi^ outnumbered IL7R^lo^ cells by 3-fold 6 weeks post transfer. This finding confirmed a superiority of memory precursor cells to survive and undergo homeostatic proliferation, but also highlighted a not-so-short-lived behavior of effector cells, as about 40 days after transfer a considerable number of remaining transferred cells was still present [19]. Furthermore, if we consider the finding that repetitive stimulation of memory T cells leads to a generally prolonged survival of effector cells, it is conceivable that MCMV-specific T_EM_ cells could become very long-lived during latency, as according to our model we might have a situation of repetitive re-stimulations of memory T cells [37,38]. Whether or not the context and frequency of antigen-presentation during CMV infection program T_EM_ cells to become long-lived, or if other factors promote prolonged persistence independent of viral antigens, remains to be addressed and is a topic that deserves attention. MCMV-specific CD8 T_EM_ cells in the lungs, liver and spleen do not express CD103, but they do so in the salivary glands (SG), where MCMV-specific CD8 T cells display a classical T_RM_ phenotype (unpublished data). It is important to note, however, that migration of CD8 T cells to SG as well their conversion to T_RM_ has been shown to be independent of local antigen presentation as it was also observed upon acute LCMV infection which does not result in local viral replication in SGs [92]. Rather, E-cadherin expression on epithelial cells and the influence of cytokines such as TGF-β [27] promote the conversion of memory T cells to a T_RM_ phenotype. However, SGs are extremely important in the context of CMV immunity as it is a relevant organ for horizontal transmission as productive virus replication persists for an extended time period in the SG. Therefore, it is conceivable that it is of interest for the host to keep a large pool of functional CD8 T cells which can limit viral dissemination. Whether these T_RM_ cells that persist in the SGs have a biological function during CMV infection is still questionable, as CD8 T cell immunity is not sufficient to control CMV in this organ but rather depends on CD4 T cells [93,94]. 

Whether other tissue-specific matrix anchor-molecules, costimulatory molecules or cytokines promote antigen-independent survival of T_EM_ in other peripheral tissues such as the lungs has not been resolved yet. 4-1BB/4-1BBL interaction has been shown to promote memory inflation during CMV infection, although the mechanism behind this effect was not investigated [95]. 4-1BB is a T cell costimulatory factor and member of the tumor necrosis factor receptor family, and has been directly implied in promoting antigen-independent survival of memory CD8 T cells by inducing Bcl-XL and Bfl-1 in an IL-15 dependent manner [96,97]. It would be interesting to investigate whether a similar mechanism also applies for CMV infection. In this line, we observed that inflationary CD8 T cells in the lungs up-regulate Bcl-2 during latency (although never reach the level of expression displayed by T_CM_), which might suggest that indeed co-stimulation or cytokines might play a so far underestimated role in promoting survival of T_EM_ cells in the periphery in the absence of antigen [82]. Another important aspect to be considered is the role of CD4 T cell help in sustaining the inflationary CD8 T cell responses. Absence of CD4 T cells has been shown to abrogate memory inflation, and although this has not been formally proven yet, it is conceivable that their function is to deliver an optimal IL-2 level to the inflationary CD8 T cells, supporting their proliferation and survival [55,90]. 

### 2.4. Memory Inflation: Friend or Foe?

It is generally accepted that maintenance of CD8 T_EM_ cells in peripheral organs is required for continuous surveillance to keep the latent virus in check, and it is therefore in the interest of the host to maintain the number and phenotype of these cells constant throughout life. As discussed above, evidence for a protective role of inflationary CD8 T cells in limiting MCMV reactivation in mice has been provided in the Balb/c model [61,98], although little is currently known about the mechanism of how CD8 T cells confer protection and whether this exceedingly large pool of CMV-specific cells is required for this, or is rather a consequence of repetitive antigen stimulation. In fact, control of primary infection and establishment/maintenance of latency are not impaired in CD8 T cell deficient mice [99,100], the reason for this being that CMV is controlled in a concerted but redundant manner by the action of CD8 T cells, CD4 T cells and NK cells. Conversely, life-long maintenance of such a high number of CMV-specific CD8 T cells may significantly contribute to immune senescence, but this is outside the scope of this review [101,102]. 

## 3. Memory Response against α-herpesviruses: Herpes Simplex Virus Type 1 (HSV-1)

Herpes-simplex virus 1 is a ubiquitous α-herpes virus that establishes latency within sensory neurons. Lifelong infection in humans is associated with spontaneous events of virus reactivation characterized by persistent viral gene expression [103,104,105]. In mice, latency is established in sensory ganglia but reactivation does not occur spontaneously and requires physical or emotional stresses [106,107]. 

### 3.1. CD8 T Cell Immunity to HSV-1

Contrary to MCMV infection, where depletion of CD8 T cells does not impair virus control and establishment of latency, absence of this subset of lymphocytes during HSV-1 infection can result in impaired termination of the replicative phase of the infection [108,109,110]. Upon ocular HSV-1 infection of mice, memory CD8 T cells selectively accumulate in latently infected sensory ganglia [111]. In C57BL/6 mice, the majority of the CD8 T cells found in the sensory ganglia are specific for a glycoprotein B-derived HSV-1 epitope (gB-_498–505_), which have been shown to block HSV-1 reactivation from latently infected sensory ganglia cultured *ex-vivo* [112], whereby a major mechanism of CD8 T cell-mediated protection is through IFN-γ secretion and release of non-cytotoxic lytic granules [111,113,114]. This non-cytotoxic mechanism of viral inactivation appears to be particularly important for the maintenance of neuronal integrity during HSV-1 infection, but could also be a general mechanism exploited by the immune system to avoid major organ damage due to persistent CD8 T cell activation during other latent viral infections. For a long time it was undoubted that migration and long-term retention of these CD8 T cells in the sensory ganglia was dependent on local and prolonged antigen exposure, especially because many of these cells expressed markers associated with recent antigen encounter, such as CD69 and granzyme B [111,115]. However, with the recent discovery of T_RM_ cells the notion is emerging that one cannot deduce whether a T cell had recent antigen exposure by solely measuring the expression of classical activation markers such as CD69. Most of the studies aiming at elucidating the antigen dependence of these locally accumulating memory T cells have been performed using the system of skin infection, which causes viral replication in epidermal cells but also a rapid dissemination to the sensory ganglia, where the virus ultimately persists in a latent form. Using this model, Gebhardt *et al.* made the observation that a population of non-migrating virus-specific CD8 T cells persisted in the skin upon resolution of the primary infection in the absence of viral antigen [22]. These cells, now classified as bona fide T_RM_, up-regulate the expression of CD103 once they enter the epidermis, are phenotypically distinct from their recirculating counterpart (high expression of CD69, low expression of CD62L) and they visibly change their morphology, becoming dendritic-shaped [116]. In contrast to memory CD4 T cells which are mainly localized the dermal layer of the skin and are highly motile and in equilibrium with the blood, T_RM_ CD8 T cells do not recirculate and can be found in the epidermis long after the control of infection. Importantly, both migration and persistence of memory T cells in the skin as well as their conversion to T_RM_ were shown to be independent of cognate antigen, but could be induced by local inflammation alone [26]. Whether CD8 T cells are completely self-sufficient in migrating to the infected sites and confer protective functions or rely on the help of other immune cells is still unclear. Using a model of vaginal HSV-2 infection in mice, Nakanishi *et al.* could show that CD8 T lymphocyte mobilization to virus-infected tissue requires the help of CD4 T cells, not only by promoting their expansion, but also by inducing local chemokine secretion *via* IFNy production, which in turn attracted effector CD8 T cells to the infected site [117]. However, T_RM_ lodgment is not always dependent on CD4 T cell help as shown for skin infection with Vaccinia virus [25]. That skin infiltration and permanent residence of memory CD8 T cells to the site of first viral contact is not just an undesired unspecific effect caused by inflammation, but has indeed a biological significance, has been recently demonstrated by the finding that these cells confer local protection against secondary infection [22]. Furthermore, these cells can also protect against de novo infection and are clearly superior to their circulating counterparts [26].

### 3.2. How the Route of Infection Impacts CD8 T Cell Memory

While specific CD8 T cells accumulate and persist at the infected sites upon local infections (shown in model of skin, vaginal and ocular infections), they don’t do so systemically, where they are present in low numbers with a resting T_CM_ phenotype [118]. However, in response to systemic HSV-1 infection, gB-_498–505_-CD8 T cells show a very comparable behavior to inflationary CD8 T cells during MCMV infection, with accumulation and maintenance of an elevated number of T_EM_ CD8 T cells during latency [119]. These cells are maintained at a constant high number throughout the life of the infected mouse and never lose their ability to secrete IFNγ and to proliferate in response to secondary infections, whereby the KLRG1^−^ population was superior to its KLRG1^+^ counterpart [119,120]. Interestingly, KLRG1^−^ and KLRG1^+^ cells increased in number to the very same extent when transferred into lymphopenic hosts, and both divided extensively. This finding is in strong contrast to what has been observed during MCMV infection, where inflationary cells completely failed to proliferate in lymphopenic hosts [56,120]. Another difference which emerged from comparable studies performed with HSV and MCMV is the antigen dependence for the maintenance of inflationary cells. Contrary to MCMV-specific T_EM_ cells, which declined with the same kinetics when transferred into naïve and immune hosts, HSV-specific cells declined in naïve recipients but not in systemically infected HSV-1 mice where they were maintained at stable number (and even slightly increased) over a period of 60 days. In addition, blocking viral replication with the antiviral compound famcyclovir had a major impact on memory inflation in HSV compared to MCMV infection, although in both cases a complete inhibition of memory inflation was seen if the treatment was started before infection [89,119]. The strong analogies in the CD8 T cell responses induced upon systemic infection with HSV-1 and CMV is indicative of similar mechanisms of latency establishment and antigen presentation patterns, and might be exploited to better understand the complex interactions between latent viruses and their hosts. 

## 4. Memory Response against γ-herpesviruses: Gamma-1 Herpes Viruses: Epstein-Barr Virus (EBV)

EBV infects about 90% of the human population. Infection usually occurs in infancy *via* salivary contacts and is often asymptomatic. However, if delayed until adolescence, primary infection can cause acute infectious mononucleosis (IM), which is characterized by prolonged high-level of virus shedding in the throat, resulting in fever, sore throat, and lymphadenopathy [121,122]. Similar to other members of the herpes-virus family, EBV persists for the life of the host as an asymptomatic latent infection in healthy individuals, whereas it can cause severe disease in immunosuppressed patients, including nasopharyngeal carcinoma, a subset of Hodgkin’s lymphomas and B cell lymphomas [123]. Primary infection is characterized by extensive virus replication in permissive cells, most likely epithelial cells of the oropharynx. 

At the same time, EBV infects B cells, inducing their transformation and proliferation which results in amplification of the latent virus reservoir [124,125]. This phase of infection involves, and requires, the expression of so called *latent proteins*, composed of six nuclear antigens, EBNAs 1, 2, 3A, 3B, 3C and -LP, and two membrane proteins, LMPs 1 and 2. In a third phase, both lytic and latent infections are controlled by the immune system, but some infected B cells down-regulate latent gene expression and become long-lived, latently infected memory B cells [126]. However, in response to physiological events such as antigen stimulation or receipt of activation/differentiation stimuli, reactivation of the latent genome resulting in low-level virus shedding provides a new antigen source, is thought to occasionally occur especially at oropharyngeal mucosal sites, which explains the frequent detection of infectious virus in the saliva of virus carriers [127,128,129]. 

### 4.1. CD8 T Cell Immunity to EBV

In both asymptomatic and acute infection leading to IM, the CD8 T cells response is dominated by cells directed towards antigens expressed during the productive replication cycle, namely: immediate-early proteins (IE), followed by early proteins (E) and finally late proteins (L), altogether referred to as *lytic cycle proteins*.

The availability of specific HLA class I-peptide tetramers allowed the longitudinal analysis, in terms of magnitude and phenotype, of CD8 T cell responses against lytic cycle and latent proteins in patients which experienced EBV infection causing acute IM [130,131,132]. These studies revealed that responses against these two classes of proteins are highly diverse in terms of their kinetics and phenotypes. CD8 T cells specific for lytic antigens expanded massively, with individual epitope responses accounting for up to 40% of the total CD8 T cell pool, and possessed a highly activated phenotype during primary infection. This massive expansion of CD8 T cells seen during primary infection appeared to be highly oligoclonal [133,134], which has led to the speculation that these cells accumulated through a bystander effect [135]. As IM symptoms resolve, the size of this cell population is reduced, leaving behind a small population of cells, which nevertheless remained CCR7^−^ CD62L^− ^and partly CD45RA^+^, a phenotype which has usually been associated with end-stage differentiated CD8 T cells. However, in contrast to CMV-specific CD8 T cells, which in addition to CD45RA^+^ are CD27^low^, EBV-specific CD45RA^+^ CD8 T cells are CD27^high^ and display high expression of the anti-apoptotic protein Bcl-2, suggesting that they might be less differentiated than their CMV counterparts. [130,136,137]. In contrast, CD8 T cells specific for EBV latent proteins were present at much lower frequencies during the acute phase of infection, yet they were maintained or even increased in numbers upon establishment of latency [131,138,139,140]. These cells remained uniformly CD45RO^+^/RA^−^ and generally expressed higher levels of CCR7 and CD62L compared to memory CD8 T cells specific for lytic antigens. Irrespective of their antigen specificity, the number of EBV-specific memory CD8 T cells appeared to be quite stable over time in the blood of carriers and patients who did recover from IM, and no sign of inflation comparable to CMV infection was observed. However, there is increasing evidence that elderly individuals harbor elevated frequencies of EBV-specific T cells [141,142,143]. Moreover, phenotypic differences between lytic and latent antigen specific CD8 T cells did not reflect functional differences, as perforin content, ability to produce IFN-γ, or ability to proliferate in response to Ag *in vitro* was comparable amongst the two sets of EBV-specific memory CD8 T cells [140]. 

### 4.2. Requirements for Migration and Local Maintenance of Memory CD8 T Cells during EBV Infection

Local accumulation of virus-specific CD8 T cells at the site of highest virus shedding, as seen during local infection by HSV, also happens during EBV infection and occurs in the tonsils, where EBV-specific CD8 T cells can reach up to 20% of the total CD8 T cell pool. The fact that CD8 T cells migrate and persist in the tonsils in response to EBV infection makes sense as both lytic and latent infections are predominantly established at oropharyngeal mucosal sites. Interestingly, migration only starts after the resolution of the lytic phase of the infection, and this is believed to be a consequence of the altered homing phenotype of effector cells during an acute infection. In fact, access to the tonsils occurs through high endothelial venules and requires the expression of CCR7 and CD62L, markers that are known to be downregulated on activated cells [144]. This also explains the dominance of CD8 T cells with specificity for latent antigens in the tonsils of patients who recovered from IM but also in healthy long-term virus carriers, as they are known to display higher level of CCR7 and CD62L compared to CD8 T cells specific for lytic antigens. Interestingly, the majority of EBV-specific cells in the tonsillar tissue express CD103, suggesting a conversion to T_RM_ phenotype, and implying an active, antigen independent maintenance of memory CD8 T cells at this site [144,145]. 

### 4.3. Gamma-2 Herpes Viruses: Murine Herpes Virus-68 (MHV-68)

MHV-68 is a natural pathogen of feral rodents and due to the genetic, virological and immunological similarities to EBV and Kaposi’s sarcoma-associated herpes virus, it is an accepted murine model for the pathogenesis of γ-herpes viruses [146]. Upon intranasal MHV-68 infection, virus replication is first observed in the lungs, before latency is established in B cells and lung epithelial cells, but also in DCs and macrophages [147,148]. The hypothesis that latency is characterized by low-level persistent virus reactivation holds also true for MHV-68 infection, whereby TLR stimulation of latently infected cells, for example through periodic exposure to pathogens, is believed to be a major mechanism driving reactivation [149,150]. 

### 4.4. CD8 T Cell Immunity to MHV-68

The induction of a functional CD8 T cell response is crucial for limiting the pathogenesis during acute infection and for contributing to control of lytic virus replication, to control the amplification of latently infected cells as well as to prevent MHV-68 reactivation from latency [151,152]. The recent identification of about twenty MHC I-restricted epitopes eliciting a CD8 T cell response and the analysis of their kinetics represented an important step towards the understanding of the regulation and function of this lymphocyte subset [153,154]. As seen for EBV, MHV-68 infection elicits a highly heterogeneous CD8 T cell response which can be subdivided into two major kinetic patterns, referred to as lytic and latent antigen-specific CD8 T cell responses. The affiliation to the one or the other subset is determined by the differential antigen availability during different phases of the infection: lytic antigens being expressed exclusively during lytic infection and latent antigens being expressed during both lytic and latency amplification phase. Lytic antigens are presented transiently by infected epithelial cells in the lungs, inducing an early peak of the specific CD8 T cells in the lungs but not in the spleen, and since infected epithelial cells are rapidly killed, lytic-antigen specific CD8 T cells show a sharp contraction after control of the lytic infection [154,155,156]. Latent antigens are per definition antigens that are presented by latently infected cells, mainly B cells, DCs and macrophages, and are expressed during both lytic infection in the lungs and the latency amplification phase in the spleen. Consistent with this, CD8 T cells specific for latent antigens peak in the spleen with a slightly delayed kinetic compared to lytic antigen specific CD8 T cells and display a slow decline [154,155]. Both populations of CD8 T cells eventually contract during latency, although those specific for latent antigens are present at slightly higher percentages compared to cells specific for lytic antigens (~0.9% *vs.* ~0.3% of CD8^+^ T cells). Notably, none of the responses analyzed shows an inflationary behavior as seen for MCMV, and no sign of exhaustion was detectable, yet the majority of antiviral CD8 T cells express KLRG1 [157], a marker for terminally differentiated cells. Consistent with the responses induced during EBV infection, CD8 T cells specific for latent antigens are predominantly CD62L^+^CD43^+^, reminiscent of a T_CM_ phenotype, while CD8 T cells specific for lytic antigens have a significantly lower expression of these markers. Nevertheless, T cells recognizing latent antigens (which display a T_CM_ -like phenotype), but not lytic antigens (displaying a T_EM_ -like phenotype), can rapidly kill target cells *in vivo,* a function that has usually been attributed to T_EM_ type of cells [155]. 

The current knowledge about the mechanism by which memory CD8 T cells are maintained during MHV-68 infection is still poor. Prolonged antigen expression during the latency amplification phase is a prerequisite for the protracted proliferation of CD8 T cells specific for latent antigens, but whether antigen is also required for maintenance of the memory T cells pool induced during long-term latency is not known. Using a transgenic mouse that indelibly marks CD8 T cells that (had) expressed Granzyme B by administration of tamoxifen, Bannard et al. showed that the MHV-68-specific CD8 T cell response is principally maintained by cells that had been primed during the initial phase of the infection, presumably by continuous turnover of the more activated T cells in the periphery, or by a continuous replacement by means of a less activated precursor subset with higher proliferation potential in secondary lymphoid organs, or supported by local expression of ‘pro-survival cytokines’ at the site of latency [158]. Infection of IL-15^−/−^ mice alters neither the primary expansion nor the maintenance of antigen-specific CD8 T cells in the spleen and peripheral tissues, arguing against a role of this cytokine for survival of memory CD8 T cells. However this study has the caveat that absence of IL-15 impairs development and survival of NK cells, which are known to be important for the clearance of other herpes virus infections, even though in separate studies it has been shown that control of acute and latent MHV-68 infection not dependent of NK cells [159,160,161,162,163]. An essential contribution of naïve CD8 T cells to sustain the memory T cell pool during latency is rather unlikely, as only naïve cells specific for one MHV-68-derived epitope were shown to be recruited during quiescent latency and some more, but not all naïve T cells get activated in response to induced reactivation from latency [164]. 

Together, the studies performed in human patients infected with EBV and in mice infected with MHV-68 indicate that like CMV, these two members of the γ-herpes virus family induce two very distinct CD8 T cell responses which differ in their kinetics, phenotype and function during latency. However, one important difference is evident between β- and γ-families of herpes virus infections, namely that the responses that dominate during the latent phase of EBV and MHV-68 infection display a T_CM-_like phenotype, which is in strong contrast to the T_EM_ phenotype of inflationary CD8 T cells during CMV latency. One explanation for the phenotypic differences during infection by γ-herpes viruses is that lytic antigens are expressed at much higher levels compared to latent antigens during latency. Another is that both classes of antigens are similarly expressed, but at different locations, rendering some CD8 T cells more exposed to antigens than others. Finally, it is possible that the massive expansion of CD8 T cells specific for lytic antigens during primary infection hampers the acquisition of typical T_CM_ markers, even in the absence of cognate antigen. 

For both infections by MCMV and EBV/MHV-68, the factors driving two different CD8 T cell kinetic patterns are still unclear. It was initially thought that different gene expression programs in different phases of the infection or in different target cells was directly linked to the generation of two CD8 T cell kinetic patterns, one dominating the acute phase of the infection and the other the latent phase of the infection. However, as already shown for MCMV, epitopes derived from the same protein do induce T cell responses of different kinetic patterns [74,154]. It is therefore more likely that the control takes place on a post-transcriptional level, probably at the level of antigen processing or MHC I-loading and surface expression. While it seems evident that expression of lytic genes *versus* latent genes in different target cells and different organs regulates the CD8 T cell responses during primary infection, it is not clear what determines the different phenotypes of lytic and latent antigen-specific CD8 T cells during latency. 

## 5. General Conclusions

The kinetics, the phenotype and the requirements for maintenance of memory CD8 T cell responses differ during acute/resolved and persistent viral infections. While antigen-independent memory CD8 T cells with a central memory phenotype predominate at late stages after resolution of acute infections, herpes virus infections induce to a significant extent populations of memory CD8 T cells with a persistent effector memory phenotype. The factors which are driving and maintaining these large populations of effector memory CD8 T cells might differ between specific herpes viruses; while repetitive antigen exposure is believed to drive memory inflation and maintenance of effector memory cells in peripheral tissues after CMV infection, skin resident memory cells after HSV-1 infection were shown to adopt this phenotype irrespective of local antigen recognition, and lytic antigen expression seems to evoke effector memory CD8 T cells during EBV and MHV-68 infection. Memory inflation, defined by continuous accumulation of effector memory CD8 T cells in peripheral tissues, is a hallmark of CMV infection but is also observed upon systemic HSV-1 infection and—in case of CMV infection—there is compelling evidence that memory inflation is driven by antigen presentation in non-hematopoietic cells.

The ability of CMV and other herpes viruses to facilitate the long-term induction and maintenance of functional effector memory CD8 T cells in peripheral tissues is not only interesting from a mechanistic point of view, but is more recently also gaining a lot of attention in the context of T cell based vaccine approaches which harness this unique property to confer local protection against heterologous virus infections in peripheral tissues.
